# Scaling up Xpert MTB/RIF technology: the costs of laboratory- *vs.* clinic-based roll-out in South Africa

**DOI:** 10.1111/j.1365-3156.2012.03028.x

**Published:** 2012-06-12

**Authors:** Kathryn Schnippel, Gesine Meyer-Rath, Lawrence Long, William MacLeod, Ian Sanne, Wendy S Stevens, Sydney Rosen

**Affiliations:** 1 Health Economics and Epidemiology Research Office, Department of Medicine, Faculty of Health Sciences, University of the WitwatersrandJohannesburg, South Africa; 2 Center for Global Health and Development, Boston UniversityBoston, MA, USA; 3 Department of Molecular Medicine and Haematology, University of the WitwatersrandJohannesburg, South Africa; 4 National Health Laboratory ServiceJohannesburg, South Africa

**Keywords:** tuberculosis, diagnostics, economics, scale-up, middle income

## Abstract

**Objective:**

The World Health Organization recommends using Xpert MTB/RIF for diagnosis of pulmonary tuberculosis (PTB), but there is little evidence on the optimal placement of Xpert instruments in public health systems. We used recent South African data to compare the cost of placing Xpert at points of TB treatment (all primary clinics and hospitals) with the cost of placement at sub-district laboratories.

**Methods:**

We estimated Xpert’s cost/test in a primary clinic pilot and in the pilot phase of the national Xpert roll-out to smear microscopy laboratories; the expected future volumes for each of 223 laboratories or 3799 points of treatment; the number and cost of Xpert instruments required and the national cost of using Xpert for PTB diagnosis for each placement scenario in 2014.

**Results:**

In 2014, South Africa will test 2.6 million TB suspects. Laboratory placement requires 274 Xpert instruments, while point-of-treatment placement requires 4020 instruments. With an Xpert cartridge price of $14.00, the cost/test is $26.54 for laboratory placement and $38.91 for point-of-treatment placement. Low test volumes and a high number of sites are the major contributors to higher point-of-treatment costs. National placement of Xpert at laboratories would cost $71 million/year; point-of-treatment placement would cost $107 million/year, 51% more.

**Conclusion:**

Placing Xpert technology at points of treatment is substantially more expensive than placing the instruments in smear microscopy laboratories. The incremental benefits of point-of-treatment placement, in terms of better patient outcomes, will have to be equally substantial to justify the additional cost to the national health budget.

## Introduction

In 2010, national tuberculosis (TB) control programmes diagnosed and notified approximately 65% of 8.8 million estimated TB cases globally and only 18% of an estimated 290 000 multidrug-resistant TB (MDR-TB) cases (World Health Organization 2011a). Earlier and improved detection of TB and MDR-TB, especially in high-burden countries, is thus an international health priority. The Xpert MTB/RIF (Xpert) assay for the GeneXpert (GX) platform (Cepheid, Sunnyvale, CA, USA), which provides both rapid and specific detection of *Mycobacterium tuberculosis* (MTB) and identification of rifampicin (RIF) drug resistance, is seen as one promising solution to this problem (Boehme *et al.* 2010; Helb *et al.* 2010; Small & Pai 2010; Van Rie *et al.* 2010). In late 2010, WHO recommended that Xpert be used as the initial diagnostic for persons suspected of MDR-TB or TB/HIV co-infection (Stop TB Department 2010).

South Africa, one of the countries most heavily burdened by both TB/HIV co-infection and MDR-TB, was among the first to begin large-scale roll-out of Xpert (World Health Organization 2011a). South Africa has extensive laboratory capacity for the detection of TB, with more than 200 active smear microscopy laboratories and an average of 1.5 liquid culture laboratories and 1.4 line probe assay (LPA) laboratories per 5 million population, exceeding WHO targets for TB diagnostic capacity (World Health Organization 2011a). Because of the high prevalence of smear-negative TB, however, diagnosis can still take weeks (Chihota *et al.* 2010), and many patients are lost to care while waiting for culture results (Boehme *et al.* 2011). In early 2011, in response to both the WHO recommendation and locally generated evidence about the potential benefits of Xpert, especially for the detection of smear-negative TB (Boehme *et al.* 2011; Scott *et al.* 2011), the National Health Laboratory Service (NHLS) of South Africa, working with the National Department of Health, developed a national roll-out plan for Xpert within South Africa (Smart 2011). Implementation of the second phase of this plan is now underway, following a successful pilot phase launched in March 2011 (Erasmus *et al.* 2011).

While the capital expense and operational complexity of the liquid culture and LPA technologies have limited placement of these services to centralized TB reference laboratories, the WHO recommendation for the placement of Xpert technology is at ‘health facility level (ideally district or sub-district level)’ (World Health Organization 2011b). The South African roll-out plan calls for the placement of Xpert instruments in more than 200 existing sub-district laboratories that currently provide smear microscopy to multiple healthcare facilities within their catchment areas, which include regional, provincial and district hospitals, 24-h community health centres and primary healthcare (PHC) clinics. Sputum samples to be tested with Xpert will be transported from the healthcare facilities to the smear microscopy laboratories, some of which are located within hospitals. Results will be returned on average within 1–3 working days but usually not on the same day as the sputum sample was collected. Thus, although the plan will greatly accelerate diagnosis of TB once samples have reached the laboratories, as well as improve the accuracy of diagnosis, it will not allow most patients to receive test results and initiate TB treatment during the visit at which they provide the first sputum sample. It may thus not reduce loss of patients to TB treatment initiation as much as placement of the instruments at point of treatment (i.e. health centres and clinics) would (Lawn *et al.* 2012).

Early reports on the Xpert MTB/RIF technology suggest that it was designed to be placed at patient-contact sites where nurses could provide treatment immediately upon diagnosis (Helb *et al.* 2010; Morris 2010; Boehme *et al.* 2011). Among the reasons for South Africa’s decision to place the Xpert technology at laboratories rather than at point of treatment were concerns about both the cost of the test and of the required improvements to the peripheral health facilities, such as air conditioning and stable electricity supply (Trébucq *et al.* 2011; World Health Organization 2011b). To assist the South African government to evaluate the potential costs of laboratory *vs.* point-of-treatment placement, we developed a cost model that uses recent data from a pilot study of Xpert at the PHC level (Page-Shipp *et al.* 2011) and the pilot phase of the South African national roll-out of Xpert (Meyer-Rath *et al.* 2012) to estimate the average cost per test performed and the total cost of rolling out Xpert for each placement scenario.

## Methods

Taking the perspective of the public sector provider, we conducted a bottom-up cost analysis of the use of Xpert for laboratory diagnosis of pulmonary TB (PTB) using data collected by the NHLS during the pilot phase (March–May 2011) of the national roll-out. We also conducted a bottom-up costing of Xpert during a pilot implementation study at an urban PHC. Further results of both cost analyses are reported elsewhere (Bistline *et al.* 2011; Meyer-Rath *et al.* 2012). We used these costs to parameterize a cost model that estimated the cost per test and the total annual cost of the laboratory *vs.* clinic placement scenarios based on 2010 test volumes at smear microscopy laboratories across South Africa. Capital costs were annualized over an estimated useful life of five years and discounted using the South African Reserve Bank average 2011 repo rate of 5.5% (South African Reserve Bank 2011). All costs were converted to USD using the 2011 average exchange rate (January–October) of 1 USD = ZAR 7.05 (Oanda.com 2011) and are reported in 2011 USD.

### Xpert test volumes, instrument placement and operations

For both scenarios, the number of Xpert tests required is based on NHLS data indicating the total volume of smear microscopy tests performed for public sector patients in 2010. The number was adjusted downward to exclude smears for extrapulmonary TB (EPTB) and TB treatment monitoring. Volume and operational parameters are summarized in Table 1. For the laboratory scenario, Xpert volumes required to replace smear microscopy for diagnosis by 2014 were calculated for each of the 223 smear microscopy laboratories. The end of 2014 was selected to allow for future growth in the number of suspects needing testing. These laboratories are spread across most districts of South Africa and are located in both urban and rural areas. The estimated Xpert volumes varied among the laboratories from six to 309 tests per day in 2014, reflecting differences in current smear microscopy volumes for each laboratory. For the point-of-treatment scenario, the 2010 smear microscopy volume was summed for each district. The estimated volume of Xpert tests required in each district were then allocated across all healthcare facilities in the district by the types of facilities present in the district, assuming that 10% of tests would be performed at provincial hospital level, 15% at district hospital level, 10% at community health centre level and the remainder at PHC level. South Africa has 3799 hospitals, primary health clinics and community health clinics that should diagnose TB and could potentially initiate TB treatment (Health Systems Trust 2010).

**Table 1:** Assumptions and sources for Xpert volumes and operational model parameters

Parameter	Value (range)	Source	Comments
Extrapulmonary TB as proportion of all TB	16%	World Health Organization 2011a	
Annual growth in TB suspect numbers	10% (0%, 6.5%)	Meyer-Rath *et al.* 2012	Based on NDOH targets for intensified HIV/TB case finding
Proportion of all smears used for treatment monitoring	8–30% (varies by district)	Health Systems Trust 2010	Calculated from TB case load for each district
Number of diagnostic smears per suspect	2	National Department of Health 2009	Based on South African TB diagnostic algorithm
Xpert error rate	Lab: 3.4% (1–3.4%); Clinic: 6.8% (2–6.8%)	Lab: NHLS pilot phase; Clinic: assumption	Errors caused by operators and environment (high temperatures, excess dust, etc) assumed to be more frequent in clinic setting
Testing days per year	Lab: 264; Clinic: 246	Lab: standard NHLS working days; Clinic: working days within a year	
Clinic down time requiring Xpert tests to be done in laboratories	2 months/year (0–6 months)	Lab: Not applicable (delays incorporated in the 1–3 day average processing time) Clinic: assumption	Days when clinic is unable to use Xpert because of cartridge or supply stock out, temperatures in excess of 30 degrees and air-conditioning not functioning due to poor maintenance or electrical outages, electrical and generator outages, staff leave or shortages, or other operational down time

NHLS, National Health Laboratory Service; PHC, primary healthcare.

The current Xpert MTB/RIF cartridge has a 2-h processing time, and GX instruments in use in South Africa have between 1 and 48 modules, which translates to a capacity of 3–256 tests per day. In the model, GX instruments were allocated to each laboratory or treatment facility according to the number of Xpert tests estimated to be required per day by the end of 2014 and the maximum number of tests that could be run per 8-h day for laboratories and 6-h day for clinics. Clinics were assigned a 6-h working day because clinic opening hours are shorter than those for laboratories, and additional time is required at the beginning of each day for the first patients to progress through clinic reception and triage before being asked to produce a sputum sample for testing. Laboratories, in contrast, would typically begin each morning testing samples which were stored in the refrigerator overnight while awaiting pickups from the clinics.

### Cost data and costing methods

The cost of the cartridge and of international freight, importation and local delivery of the cartridge were assumed to be the same regardless of the placement. Other costs were varied both across the placement scenarios and according to the estimated volume of the tests at the site. Estimation methods and sources of the cost components are summarized in Table 2. Costs per site, instrument or day were translated into costs per test according to the national average volume of tests for those instruments.

**Table 2:** Model cost assumptions and sources

Cost	Value (range)	Source	Comments
Recurrent costs			
Xpert MTB/RIF cartridge	$14.00 ($10.72–$16.86)	Published prices from manufacturer (Cepheid)	Prices dependent on cumulative global volume of cartridges procured, Stop TB Department 2010. Price assumed at $14.00 in 2014, based on expected procurement volumes by South Africa 2012–2013
Cartridge procurement	$2.68 ($2.05–$3.23)	Quotation from local supplier	Inclusive of air freight, customs and importation, insurance, and local delivery charges. Varies with cartridge cost
Module calibration	$496/module, exclusive of labor and travel	Quotation from local supplier	Module calibration required after every 2000 tests or after 1 year, whichever occurs first. ‘Swap pack‘calibration method used
Sample consumables	See Table 4 for per test costs	Lab: NHLS pilot phase expenditure; Clinic: PHC pilot study	Includes gloves, disinfectant, and N-95 masks (per day) and sputum collection bottles, request forms, and specimen bags (per test)
Salaries	Lab: Technician at $24 454/year; Clinic: Staff nurse at $28 450/year	Lab: NHLS pilot expenditure; Clinic: NDOH salary scales	Lab: laboratory technician (1 year laboratory training) Clinic: staff nurse (2 years nursing school)
Operator staff time per test	Lab: 0.2 h/test Clinic: 0.25 h/test	Lab: NHLS pilot phase; Clinic: PHC pilot study	Allocated at ‘hands-on’ time per test for GX1-GX12 100% effort for GX16 instruments and above
Management staff salaries	Lab: Laboratory manager at $52 817/year; Clinic: $55 516/year	Lab: NHLS pilot expenditure; Clinic: NDOH salary scales	2% level of effort
Transport of supplies and/or samples	Lab: 8% of cartridge + consumables cost; Clinic: 3% cartridge + consumables cost	Lab: NHLS pilot phase Clinic: Quotation	
External quality assessment	See Table 4 for per test costs	NHLS pilot phase expenditure	Three times per year for each module, following calibration
Training (2 days on-site)	See Table 4 for per test costs	NHLS pilot phase expenditure	Includes trainer, travel, meals, accommodation, training materials. Lab: every other year Clinic: every year (due to higher staff rotation)
Overhead cost	Lab: 12% of other direct test costs; Clinic: see Table 4 for per test costs	Lab: NHLS pilot phase; Clinic: PHC pilot study	Clinic: Includes electricity, water, medical waste disposal, security services, cleaning services, and space (rent). Expenses allocated according to the proportion of total space required for each type of instrument (Cepheid n.d.).
Capital costs			
GX instruments	GX-IV with 4 modules and desktop computer at $17 000	GX-IV: Published prices from manufacturer (Cepheid); Other GX costs: quotation from local supplier	Includes international freight, customs and importation, insurance, uninterrupted power supply unit, desktop computer, printer, barcode reader, installation and delivery.
Renovations	See Table 4 (Other equipment)	Lab: NHLS pilot phase expenditure; Clinic: PHC pilot study expenditure	Includes minor renovations for shelves or security, air-conditioning, network points, and generator installation. Lab: additional extensive renovations for the GX48 because of large footprint and excess weight
Data management system	See Table 4 (Other equipment)	NHLS pilot phase expenditure	Included for any instrument using a GX16 case as well as GX48
Generator	Lab: 85% existing coverage Clinic: 0% existing coverage	Published local prices for generators; Lab: NHLS pilot phase for coverage Clinic: Assumption for coverage	Based on Cepheid Xpert specifications (Cepheid n.d.). Generator back-up capacity not calculated to power air conditioning
Refrigerator for sample storage	Lab: 85% existing coverage Clinic: Not included	Lab: NHLS pilot for coverage; Published local prices for refrigerators	Clinics would not need to store samples as providing the service while patient waits
Useful life of equipment	5 years (3–8 years)	Assumption	

NHLS, National Health Laboratory Service; PHC, primary healthcare.

Capital costs, as incurred by the NHLS in the pilot roll-out phase, include the GX instruments (case and modules), desktop computers, printers, uninterrupted power supply systems, barcode readers, air conditioners, renovations, project management time for the roll-out and installation, data management systems, backup generators and refrigerators for sample storage. Bio-safety equipment, such as a biohazard hood, was excluded for point-of-treatment placement because of the low risk of aerosols in using Xpert (Banada *et al.* 2010) and for laboratory placement because of existing capacity.

The recurrent cost per Xpert test includes the Xpert MTB/RIF cartridge, cartridge procurement, module calibration, test consumables, labour, external quality assessment and operator training, transport of samples and consumables, and operating overheads. Required operator time was based on the bottom-up costing of the NHLS pilot roll-out and the PHC pilot for the laboratory and clinic placement scenarios, respectively. A 2-day on-site training for GX operators was included biannually for laboratory technicians and annually for clinic nurses because of turnover and rotation of staff within clinics. Per site training costs incurred by the NHLS in the pilot roll-out phase were applied to both scenarios. Annual quality assurance site visits by NHLS staff was also assumed for both scenarios, mirroring systems established and costs incurred in the NHLS pilot phase. Sample transport from clinics to the laboratories was included in the laboratory placement at the standard NHLS markup of 8% per test. For clinic placement, a charge of 3% for the transport of cartridges and sputum collection supplies from a district depot to the peripheral facilities was included. Overhead costs were included at the standard NHLS per test 12% markup for the laboratory placement. For the clinic placement scenario, overhead costs included electricity, generator fuel, water, medical waste disposal, security and cleaning services, and required clinic space.

The Xpert error rate from the NHLS pilot roll-out phase was used as the baseline laboratory error rate. Clinic placement was assumed to have twice as many errors as the laboratory scenario because of less experienced operators, off-site (and therefore less frequent) quality assurance and management, and greater environmental instability especially in terms of maintaining temperatures within the manufacturers’ recommended operating range of less than 30 degrees Celsius (World Health Organization 2011b). We assumed that the same factors would also lead to a need for clinic staff to access laboratory-based Xpert for a proportion of tests throughout the year.

### Sensitivity analysis

A number of input parameters were varied in sensitivity analysis. The base case for both scenarios assumes a cost per Xpert cartridge of $14.00 in 2014, annual growth in TB suspects of 10%, a useful life of equipment of 5 years, an Xpert error rate of 3.4% for laboratory placement and 6.8% for clinic placement and the need to access a laboratory-based Xpert instrument for on average 2 months each year when clinics would be unable to provide the service. For the cartridges, the current price of $16.86 available to high-burden countries and the future discounted price of $10.72 were both considered as alternatives, as the price is dependent on the cumulative international volume of cartridges procured (Stop TB Department 2010). The number of PTB suspects was varied to consider a scenario of no growth and one where the suspects increase by 6.5% annually, according to the assumption used in the Planning and Budgeting for TB Control Model for South Africa (Stop TB Department 2006). The useful life of the equipment was varied from 3 to 8 years, but was kept the same for both placement options. Scenarios were considered in which the error rate of 3.4% was the same for both placements and in which both rates were decreased, to 1% for laboratories and 2% for point of treatment, because of expected improvements to the Xpert MTB/RIF cartridge. Finally, the proportion of tests that would have to be performed in laboratories under point-of-treatment placement was varied from 0% (i.e. all tests performed in clinics, no ‘down’ time) to 50%.

## Results

National scale-up to existing smear microscopy laboratories at sub-district level will require 274 Xpert instruments ranging in size from GX1 to GX48, with a total of 2739 modules. These will cost $16 million to procure. Scale-up to points of treatment will require 4020 instruments (GX1–GX16, a total of 5056 modules) which will cost $41 million to procure, 2.5 times more than the procurement of instruments for laboratory placement. Table 3 details the estimated need for GX instruments and the capital costs of placement.

**Table 3:** Capital costs* of placement, by instrument type (2011 USD)

GX instrument†	Laboratory placement scenario		Point-of-treatment placement scenario	
	
	Number of instruments	Total capital cost (2011 USD)	Number of instruments	Total capital cost (2011 USD)
GX1‡ (GX-IV case)	4	$40 142	3533	$42 796 050
GX2 (GX-IV case)	12	$174 035	294	$4 801 101
GX3 (GX-IV case)	29	$550 143	89	$1 817 305
GX4 (GX-IV case)	17	$366 887	56	$1 303 122
GX8 (GX-XVI case)	85	$5 112 351	36	$2 260 103
GX12 (GX-XVI case)	47	$3 590 829	9	$714 712
GX16 (GX-XVI case)	79	$7 313 096	3	$286 748
GX48 (GX-Infinity case)	1	$399 812	0	–
Total	274	$17 547 295	4 020	$53 979 142
Total number of GX modules	2739		5056	

* Capital costs inclusive of GX instrument, desktop computer, uninterrupted power supply, desktop printer, generator, refrigerator, data management information system, air conditioning, renovations, and delivery, installation and roll-out of the above.

† GX instrument and module costs are from quotations from local supplier, August 2011. GX-IV case at ‘compassionate’ pricing level. Other cases at price negotiated between NHLS and local supplier.

‡ GX-I case not eligible for discounted ‘compassionate’ pricing; therefore costs given here are for a GX-IV case equipped with one module, which as per local supplier quotation is less expensive than the GX-I.

In 2014, at full national scale, the total cost per test performed is $26.54 in the laboratory scenario and $38.91 in the point-of-treatment scenario, an additional $12.37 or 47%. The per-test cost for both scenarios is driven by the cost of the cartridge, assumed to be $14.00 in 2014 and comprising 53% and 36% of total per-test costs in the laboratory and point-of-treatment placement scenarios, respectively. Three items make major contributions to the difference in the cost per test between the scenarios, as indicated in Table 4: instrument procurement, external quality assessment and training, and labour. The cost of the Xpert instruments comprises just 6% and 11% of the total cost per test in the laboratory and point-of-treatment scenarios, respectively, but contributes 20% of the difference between scenarios. On-site training and quality assurance delivered to more than 4000 sites comprises 10% of point-of-treatment test costs while remaining a negligible component of laboratory placement, thus contributing 30% of the difference between the scenarios. Efficiencies in sample preparation and operation for the larger scale (GX16 and GX48) instruments lead to lower labour cost per test in the laboratory scenario and account for 20% of the difference in the per-test cost. The breakdown of the cost per Xpert test in each scenario is shown in Table 4.

**Table 4:** 2014 Cost per successful Xpert MTB/RIF test, by scenario (2011 USD)

Cost component	Cost per test (% of total)		Additional cost for point of treatment (% of total difference)

	Laboratory placement	Point-of-treatment placement	
Recurrent costs			
Xpert MTB/RIF cartridge	$14.00 (53)	$14.00 (36)	$0.00
Cartridge procurement	$2.68 (10)	$2.68 (7)	$0.00
Labor	$2.90 (11)	$5.35 (14)	$2.45 (20)
Overhead operating costs	$2.68 (10)	$4.25 (11)	$1.57 (13)
Sample, supplies transport	$1.36 (5)	$0.68 (2)	−$0.68 (−5)
Module calibration	$0.60 (2)	$1.47 (4)	$0.87 (7)
Consumables	$0.36 (1)	$1.20 (3)	$0.84 (7)
Quality assessment and training	$0.15 (1)	$3.87 (10)	$3.72 (30)
Capital costs			
GX instruments	$1.66 (6)	$4.16 (11)	$2.50 (20)
Other equipment, renovations	$0.15 (1)	$1.25 (3)	$1.10 (9)
Totals (% additional)			
Total cost per test	$26.54	$38.91	$12.37 (+47)
Total cartridges procured to test 2.6 million TB suspects in 2014	2.7 million	2.8 million	0.1 million (+3)
Total annual cost (2011 USD) in 2014	$71 million	$107 million	$36 million (+51)

In 2014, South Africa will use Xpert as the first-line diagnostic for testing 2.6 million PTB suspects. For this volume, laboratory placement would cost $71 million per year. Point-of-treatment placement would cost $107 million per year, 51% more than laboratory placement.

### Sensitivity analysis

In sensitivity analysis, presented in Table 5, we found that varying core assumptions leads to an annual cost for the point-of-treatment scenario that is 43–65% higher than laboratory placement. Apart from the cost per cartridge, cost per test in the laboratory placement scenario was less sensitive to changes in the core assumptions, varying by −2% to +5%. Cost per test in the point-of-treatment scenario varied by −5% to +8% as factors affecting the utilization of the instruments changed. Because the capital per-test cost is 3 times higher in the point-of-treatment scenario, differences in per-test cost are sensitive to assumptions about the estimated annual growth in TB suspects and the expected useful life of equipment.

**Table 5:** Results of sensitivity analysis: 2014 Cost per test and annual costs, by scenario (2011 USD)

	Laboratory placement		Point of treatment placement		Additional annual cost for point-of-treatment (%)
	
	Cost per test	Annual cost	Cost per test	Annual cost	
Base case*	$26.54	$71 045 331	$38.91	$107 282 983	$36 237 652 (51)
Growth in suspect population					
0% annual growth	$26.40	$48 716 554	$42.18	$80 171 695	$31 455 141 (65)
6.5 % annual growth	$26.41	$62 268 732	$39.97	$97 047 560	$34 778 828 (56)
Clinic service gaps requiring laboratory back-up					
No outages	$26.54	$71 045 331	$38.01	$105 356 422	$34 311 091 (48)
Average 6 months/year	$26.54	$71 045 331	$40.12	$109 287 682	$38 242 351 (54)
Error rate					
Clinic same as lab (3.4%)	$26.54	$71 045 331	$39.44	$105 784 390	$34 739 059 (49)
Both reduced (1%, 2%)	$26.52	$69 316 411	$39.66	$104 770 496	$35 454 085 (51)
Useful life of GX and other equipment					
3 years	$27.74	$74 229 660	$42.10	$116 018 843	$41 789 183 (56)
8 years	$25.89	$69 279 232	$37.14	$102 350 115	$33 070 883 (48)
Future discount on Xpert MTB/RIF cartridges (current international price $16.86)					
No discount ($16.86)	$30.64	$81 989 790	$42.52	$117 176 276	$35 186 486 (43)
Discounted price of $10.72	$21.87	$58 522 086	$34.78	$95 846 446	$37 324 360 (64)

* Base case: 2.6 million suspects, 10% growth in suspects per year; 2 months service outage in clinics per year; 3.4% error in laboratories and 6.8% error in clinics; 5 years useful life of GX instruments; $14.00 cartridge cost.

## Discussion

The literature on the use of Xpert MTB/RIF and other new diagnostic technologies typically referred to as ‘point of care’ has only recently begun to consider exactly where the technologies should be placed (Trébucq *et al.* 2011). In this analysis of the cost of the national rollout of Xpert for first-line PTB diagnosis in South Africa, based on locally generated data on test volumes and costs, we estimated that truly decentralized placement at the point of TB treatment (clinics and hospitals) is approximately 51% more expensive than placement at sub-district laboratories. This additional cost of $36 million would represent a 17% increase in the overall estimated $218 million TB control budget for South Africa for 2011 (World Health Organization 2011a). The additional cost of point-of-treatment placement is attributable to two main factors. First, many more sites must be initially capacitated with GX instruments, equipment and trained staff. Second, the lower volumes of tests conducted per day in clinics diminish the technical and economic efficiency with which each instrument can be operated.

Despite its higher cost, placing Xpert in at point of treatment offers the potential to reduce the loss of patients before initiation of TB treatment. Healthcare facilities with access to both TB treatment and on-site Xpert diagnostic capability could potentially have TB suspects provide a sputum sample, receive test results and initiate TB treatment on the same day. Xpert also rapidly diagnoses RIF resistance, an important marker for MDR-TB. Current South African MDR-TB guidelines indicate that only a limited number of capacitated hospitals should initiate MDR-TB treatment. Thus, while the placement of Xpert technology at health facilities may reduce delays in MDR-TB treatment compared with the laboratory placement, it is unlikely that patients with Xpert-detected RIF resistance will be able to initiate MDR-TB treatment on the same day in either scenario. Further economic analysis, including research that incorporates treatment outcomes and analysis that takes into account the costs and benefits of the scenarios to patients, the health system and society for both drug-sensitive and drug-resistant TB, is needed to fully appreciate the differences between the scenarios.

The per-test cost of Xpert at both laboratory and point of treatment reported here are higher than previously reported estimates from South Africa (Theron *et al.* 2011; Vassall *et al.* 2011). The local prices for procurement of the cartridges and instruments used in our analysis were higher than those used in other estimates. Also, this analysis does not assume the exclusive use of GX4 instruments or average volumes, as was the case for the previous estimates, but rather uses the actual range of GX instruments anticipated to be in use and current daily TB test volumes from South Africa. Finally, this analysis was designed to include the costs of the overall roll-out of the Xpert technology at a national scale, which we show can be a significant component of the total cost per test for a national TB control programme.

Although the analysis reported here is based on primary data from South Africa, it has several limitations. First, unit cost estimates are based on small samples and early pilot studies which may not reflect costs at scale. Second, current smear microscopy volumes may not accurately estimate the need for Xpert, even if allowing for future growth in the number of suspects. Adjustments made to current volumes to exclude smears for diagnosing EPTB may underestimate the volume of Xpert tests that will be required if Xpert becomes the diagnostic of choice for EPTB (Vadwai *et al.* 2011) and/or paediatric TB (Nicol *et al.* 2011) as well. Third, the analysis does not take into account the time required to implement either scenario, but rather assumes that Xpert provision will reach full scale immediately. Placing instruments and supporting their use at more than 4000 facilities for the point-of-treatment scenario, most of which lack existing laboratory infrastructure, will be a far more complicated undertaking than placing them at just over 200 laboratories and may limit access to this rapid diagnostic and its benefits for a far longer period than the roll-out to laboratories. We attempted to capture this in our cost estimates by including additional training and supervision time as well as laboratory backup of clinic testing capacity, but this might not capture the full difference in operational complexity. Fourth, the analysis is based on the cost of Xpert within the current diagnostic algorithm for South Africa. Alternative diagnostic algorithms, such as using Xpert only for smear-negative, HIV-infected TB suspects (Page-Shipp *et al*. 2011; Theron *et al.* 2011) would have to be analysed according to their impact on test volumes. Finally, the comparison presented here assumes an ‘either/or’ decision with regard to Xpert placement – either in sub-district laboratories or at point of treatment, but not both. A combination of laboratory and point-of-treatment placement may be preferable and is likely to be the strategy ultimately adopted by South Africa.

The results of the analysis pertain to South Africa and may not be readily generalizable to other high-TB-burden countries. Unlike many other low- and middle-income countries, South Africa has a strong infrastructure of existing sub-district laboratories and an excellent transport network that allows for efficient collection and processing of samples. The potential benefits of point-of-treatment diagnosis may be greater in countries where there are no or very few laboratories and where patient travel distances to clinics are longer; though, the challenges and costs of clinic placement in such settings may also be greater. Although the specific results of this analysis may not be readily transferrable to other countries, the issues it examines, such as the relationship between the cost per Xpert test and the volume of tests performed, will be of relevance to all countries that are considering its use.

Despite the limitations described above, we conclude from this analysis that point-of-treatment placement of Xpert technology is ultimately more expensive per test because of the inadequacy of existing clinic infrastructure and low test volumes in each health facility. A substantial increase in treatment uptake, large improvement in treatment outcomes and/or significant cost savings to patients would be needed to justify the higher costs of this placement. While access to Xpert may indeed facilitate achieving these goals, other health system investments may also be needed to secure them. Given the resource constraints faced by most high-TB-burden countries, lower-cost interventions to reduce loss to TB treatment initiation, such as reducing other causes of delay in diagnosis and treatment (Sreeramareddy *et al.* 2009) and continued efforts to develop point-of-treatment tests (Dorman *et al.* 2012), combined with laboratory-based access to Xpert technology, may be more cost-effective investments than point-of-treatment placement of Xpert instruments. It is important that this question be investigated in a range of settings throughout high-TB-burden countries.

## References

[b1] Banada PP, Sivasubramani SK, Blakemore R (2010). Containment of bioaerosol infection risk by the Xpert MTB/RIF assay and its applicability to point-of-care settings. Journal of Clinical Microbiology.

[b2] Bistline K, Van Rie A, Page-Shipp L, Basset J, Sanne I (2011). Cost of Xpert MTB/RIF for smear-negative TB suspects at primary care clinic in Johannesburg. International Journal of Tuberculosis and Lung Disease.

[b3] Boehme CC, Nabeta P, Hillemann D (2010). Rapid molecular detection of tuberculosis and rifampin resistance. New England Journal of Medicine.

[b4] Boehme CC, Nicol MP, Nabeta P (2011). Feasibility, diagnostic accuracy, and effectiveness of decentralised use of the Xpert MTB/RIF test for diagnosis of tuberculosis and multidrug resistance: a multicentre implementation study. Lancet.

[b5] Cepheid http://www.genexpert.com/pdfs/GeneXpert.

[b6] Chihota VN, Grant AD, Fielding K (2010). Liquid vs. solid culture for tuberculosis: performance and cost in a resource-constrained setting. International Journal of Tuberculosis and Lung Disease.

[b7] Dorman S, Manabe Y, Nicol M, Nakiyingi L, Moodley M, Zemanay W (2012).

[b8] Erasmus LK, Coetzee GJ, Stevens WS (2011). Scale up of Xpert MTB/RIF from the national laboratory perspective: issues and challenges. International Journal of Tuberculosis and Lung Disease.

[b9] Health Systems Trust (2010). The District Health Barometer 2008/09.

[b10] Helb D, Jones M, Story E (2010). Rapid detection of Mycobacterium tuberculosis and Rifampin resistance by use of on-demand, near-patient technology. Journal of Clinical Microbiology.

[b11] Lawn SD, Kerkhoff AD, Wood R (2012). Location of Xpert® MTB/RIF in centralized laboratories in South Africa undermines potential impact. International Journal of Tuberculosis and Lung Disease.

[b12] Meyer-Rath G, Schnippel K, Long L (2012). The impact and cost of scaling up GeneXpert MTB/RIF in South Africa. PLoS One.

[b13] Morris K (2010). Xpert TB diagnostic highlights gap in point-of-care pipeline. The Lancet Infectious Diseases.

[b14] National Department of Health (2009). South African National Tuberculosis Guidelines.

[b15] Nicol MP, Workman L, Isaacs W (2011). Accuracy of the Xpert MTB/RIF test for the diagnosis of pulmonary tuberculosis in children admitted to hospital in Cape Town, South Africa: a descriptive study. The Lancet Infectious Diseases.

[b16] Oanda.com (2011). http://www.oanda.com/currency/average.

[b17] Page-Shipp L, Dansey H, Basset J (2011). Point of care Xpert MTB/RIF for smear-negative TB diagnosis at a primary care clinic in South Africa. International Journal of Tuberculosis and Lung Disease.

[b18] Scott LE, Mccarthy K, Gous N (2011). Comparison of Xpert MTB/RIF with other nucleic acid technologies for diagnosing pulmonary tuberculosis in a high HIV prevalence setting: a prospective study. PLoS Medicine.

[b19] Small PM, Pai M (2010). Tuberculosis diagnosis — time for a game change. New England Journal of Medicine.

[b20] Smart T (2011). http://www.aidsmap.com/GeneXpert-to-be-rolled-out-as-first-line-diagnostic-for-TB-in-South-Africa/page/1746803/.

[b21] South African Reserve Bank (2011). http://www.resbank.co.za/Research/Rates/Pages/SelectedHistoricalExchangeAndInterestRates.aspx.

[b22] Sreeramareddy CT, Panduru KV, Menten J, Van den Ende J (2009). Time delays in diagnosis of pulmonary tuberculosis: a systematic review of literature. BMC Infectious Diseases.

[b23] Stop TB Department (2006). http://www.who.int/tb/dots/planning_budgeting_tool/en/.

[b24] Stop TB Department (2010). Roadmap for Rolling Out Xpert MTB/RIF for Rapid Diagnosis of TB and MDR-TB.

[b25] Theron G, Pooran A, Peter J (2011). Adjunct TB tests, when combined with Xpert MTB/RIF, improve accuracy and the cost of diagnosis in a resource-poor setting?. ERJ.

[b26] Trébucq A, Enarson DA, Chiang CY (2011). Xpert® MTB/RIF for national tuberculosis programmes in low-income countries: when, where and how?. International Journal of Tuberculosis and Lung Disease.

[b27] Vadwai V, Boehme CC, Nabeta P, Shetty A, Alland D, Rodrigues C (2011). Xpert MTB/RIF: a new pillar in diagnosis of extrapulmonary tuberculosis?. Journal of Clinical Microbiology.

[b28] Van Rie A, Page-Shipp L, Scott L, Sanne I, Stevens W (2010). Xpert ® MTB/RIF for point-of- care diagnosis of TB in high-HIV burden, resource-limited countries: hype or hope?. Expert Review of Molecular Diagnosis.

[b29] Vassall A, van Kampen S, Sohn H (2011). Rapid diagnosis of tuberculosis with the Xpert MTB/RIF assay in high burden countries: a cost-effectiveness analysis. PLoS Medicine.

[b30] World Health Organization (2011a). Global Tuberculosis Control.

[b31] World Health Organization (2011b). Rapid Implementation of the Xpert MTB/RIF Diagnostic test: Technical and Operational “How-to”; Practical Considerations. World Health (WHO/HTM/TB.

